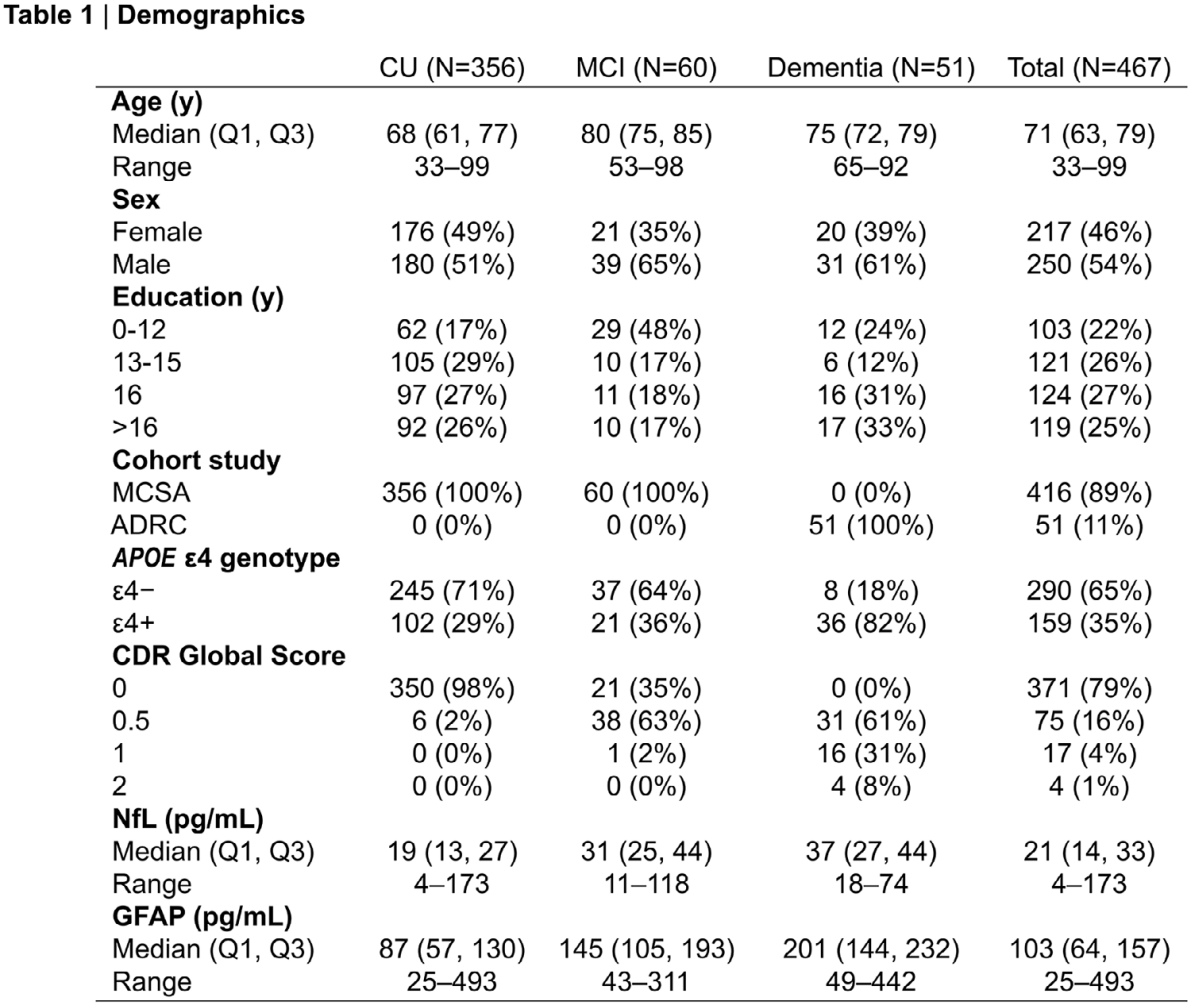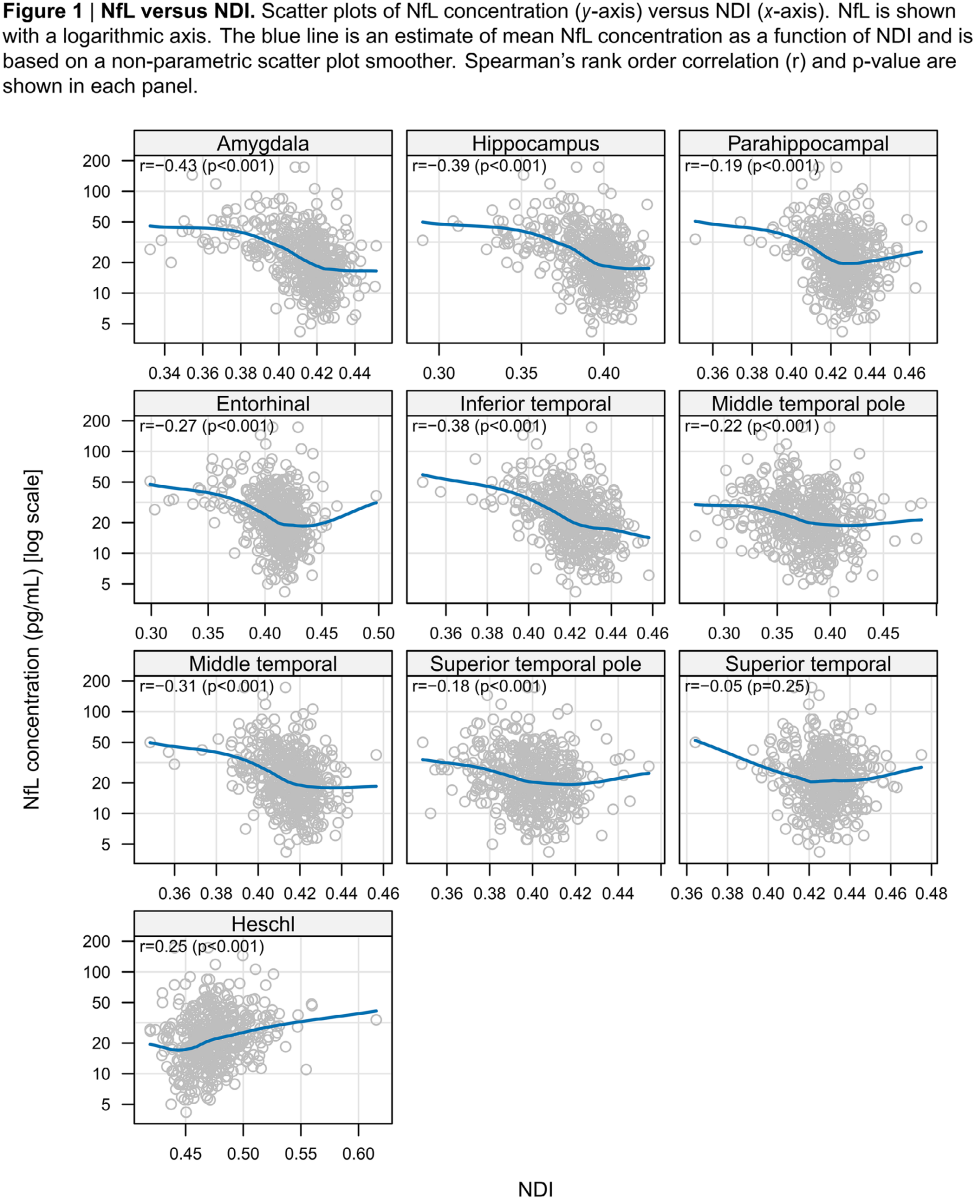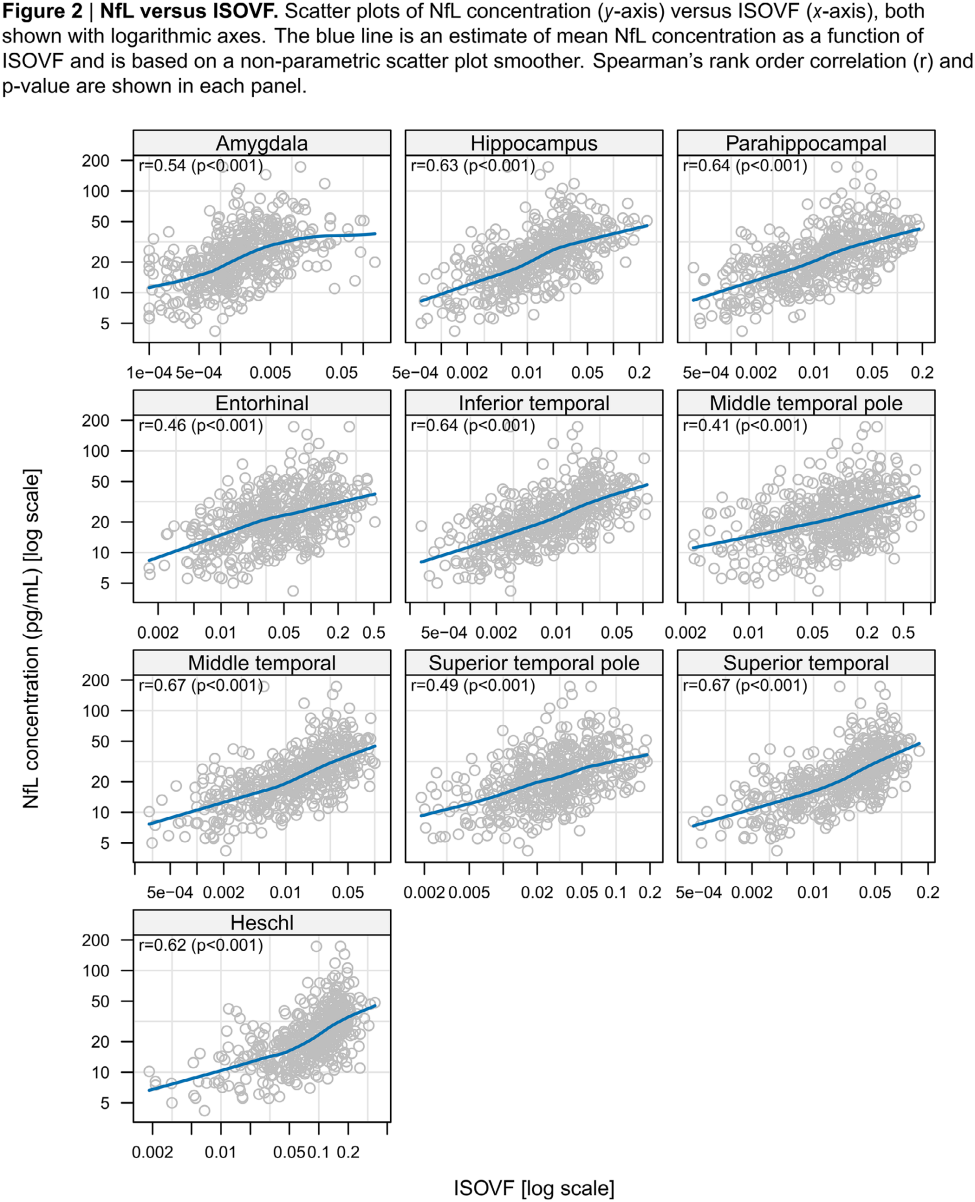# Association between temporal lobe cortical NODDI measures and plasma biomarkers

**DOI:** 10.1002/alz70862_109858

**Published:** 2025-12-23

**Authors:** Jay J Pillai, Robert I. Reid, Stephen D. Weigand, Petrice M Cogswell, Prashanthi Vemuri, Alicia Algeciras‐Schimnich, David S. Knopman, Jonathan Graff Radford, Ronald Petersen, Clifford R. Jack

**Affiliations:** ^1^ Mayo Clinic, Rochester, MN USA; ^2^ Department of Radiology, Mayo Clinic, Rochester, MN USA; ^3^ Department of Neurology, Mayo Clinic, Rochester, MN USA

## Abstract

**Background:**

Plasma neurofilament light chains (NfL) and glial fibrillary acidic protein (GFAP) are blood‐based biomarkers of axonal injury and astrocytic activation, respectively, that are elevated in those with Alzheimer’s disease (AD) and mild cognitive impairment (MCI) relative to cognitively unimpaired (CU) individuals. Neurite orientation dispersion and density imaging (NODDI) is a multishell diffusion MRI method capable of evaluating tissue microstructure at the axonal and dendritic level. We evaluated associations between temporal cortex orientation dispersion index (ODI), neurite density index (NDI) and isotropic volume fraction (ISOVF) and plasma NfL and GFAP concentrations.

**Method:**

We included Mayo Clinic Study of Aging (MCSA) participants (ages 60‐95 years) with diagnoses or CU (*n* = 356) or MCI (*n* = 60), and Alzheimer’s Dementia Research Center (ADRC) participants with dementia (*n* = 51). NODDI imaging was obtained on a 3T Siemens Prisma scanner with 2 mm isotropic resolution. NfL and GFAP were measured using the Simoa® Neurology 4‐Plex E Advantage kit. We fit separate linear regression models within each MCALT_ADIR122 cortical ROI with regional NODDI as the primary predictor and log‐transformed plasma concentration as the outcome. Models were adjusted for age, sex, BMI, cortical thickness (or hippocampal & amygdalar volume) and total intracranial volume.

**Result:**

The Table displays demographic data. Figures 1 & 2 show the relationship of NDI and ISOVF measures to plasma NfL concentrations across participants in individual ROIs (similar findings were noted with GFAP). A significant inverse relationship between NfL concentrations and NDI was observed in hippocampus, amygdala, entorhinal cortex, parahippocampal gyrus as well as all temporal neocortical regions except the superior temporal gyrus (*p* <0.001) (Figure 1), whereas higher NfL concentrations were associated with higher ISOVF in all regions (*p* <0.001) (Figure 2). Fewer significant associations were also seen with ODI. After adjusting for age, cortical thickness/volume and other confounds, significant associations with NDI remain, particularly in hippocampal and amygdalar regions.

**Conclusion:**

NODDI‐derived measures (NDI and ISOVF) within the temporal lobe cortical regions demonstrate significant associations with plasma biomarkers of axonal and astrocytic dysfunction that are elevated during cognitive decline. This association provides preliminary validation of the utility of NODDI in assessing cortical microstructural changes associated with cognitive decline.